# mSphere of Influence: MmuPV1—a dual tropic papillomavirus, red herring, or novel insight into HPV pathogenesis

**DOI:** 10.1128/msphere.00177-24

**Published:** 2024-06-26

**Authors:** James Romero-Masters

**Affiliations:** 1 Department of Biomedical Sciences and Pathobiology, Virginia-Maryland College of Veterinary Medicine, Virginia Tech, Blacksburg, Virginia, USA; Virginia Commonwealth University, Richmond, Virginia, USA

**Keywords:** human papillomavirus, MmuPV1, pathogenesis, mouse models

## Abstract

James Romero-Masters works in the field of tumor virology, focusing on the role of the human papillomavirus oncogenes in pathogenesis. In this mSphere of Influence article, they reflect on how the article “Mouse papillomavirus infection persists in mucosal tissues of an immunocompetent mouse strain and progresses to cancer” impacted them, informing their research strategies, and what it means for the mouse papillomavirus model.

## COMMENTARY

Human tumor viruses contribute to at least 10% of all human cancers globally and half of those cancers can be attributed to a single virus family, human papillomaviruses (HPVs) (1). While the HPV vaccine has provided a key tool in combating papillomavirus-associated cancers, the uptake of the HPV vaccine in the United States is limited and falls below the goals set forth by the WHO’s cervical cancer elimination program (2). A missing tool for combating HPV-associated disease and cancer is the development of effective anti-viral therapies for persistent HPV infections, which is a prerequisite for HPV-driven cancers. A key barrier to the development of antiviral therapies for persistent HPV infections is the species specificity of papillomaviruses which prevents the study of HPV infection in a preclinical animal model. A seminal study in the development of an infection-based mouse model for HPV pathogenesis is “Mouse papillomavirus infection persists in mucosal tissues of an immunocompetent mouse strain and progresses to cancer” by Cladel et al. (3). The research in this article examines how the murine papillomavirus, MmuPV1, which was discovered in 2011, is capable of infecting the mucosal tissues of immunocompetent mice (3, 4). The work done in this study complements their previous observations in immunocompromised animals where they found MmuPV1 caused secondary infections (not experimentally infected) in mucosal tissues (5). Thus, MmuPV1 appears to have a dual tissue tropism and causes disease in both cutaneous and mucosal tissues.

In Cladel et al., they infected both immunocompromised and immunocompetent mouse strains with MmuPV1 at multiple sites including the skin, cervicovaginal tract, oral cavity, and anal tract (3). To determine if strains were susceptible to infection, they examined mice for disease development on the skin and used longitudinal methods such as gavage to tract infection at mucosal sites. To validate their observations, they performed histological analysis to determine disease severity and viral presence using *in situ* hybridization assays. Through their analysis, they found that MmuPV1 infects and causes disease at mucosal sites, in particular the cervicovaginal tract. These findings have been well corroborated by other labs.

The studies performed by Cladel et al. demonstrate that MmuPV1 displays a dual cutaneous/mucosal tropism, which is an uncommon property for papillomaviruses in general (3). This property is of further interest because MmuPV1 genetically is more related to cutaneous HPVs in genomic structure and shares certain biochemical properties with HPVs that cause cutaneous disease (6). Therefore, the dual tropism of MmuPV1 raises a number of interesting and provocative questions, especially in regard to its ability to cause mucosal disease. The HPV E6 and E7 oncogenes contribute directly to virally induced carcinogenesis both for mucosal and cutaneous HPVs. The same is true for MmuPV1. There are differences and similarities between the biochemical activities of mucosal and cutaneous HPV E6 and E7 proteins that are implicated in their roles in cancer. Mucosal HPV E6 proteins preferentially bind to the LXXLL motif-containing binding partner, E6AP, a cellular ubiquitin ligase, that promotes proteasomal degradation of other cellular factors that associate with the E6 protein, including the tumor suppressor p53 (7–9). In contrast, cutaneous HPV E6 proteins preferentially bind to the LXXLL motif-containing binding partner, MAML1, to inhibit tumor suppressive NOTCH signaling (7, 10). MmuPV1 E6, like cutaneous E6 proteins, binds to MAML1 and does not bind or induce degradation of p53 (11). In the case of E7, distinctions are less clear. Like most HPV E7 proteins, MmuPV1 E7 can bind to the cellular tumor suppressor, Rb, which is a key cell cycle regulator (12, 13). However, unlike many mucosal and cutaneous HPV E7 genes, MmuPV1 E7 does not drive cell proliferation. Rather, MmuPV1 E6 is the main driver of cell proliferation (14). A reasonable hypothesis for this divergence in MmuPV1 E7 biology could be linked to MmuPV1 E7 interacting with amino acids in the c-terminal domain of pRB instead of binding to the pocket domain of pRB through an LXCXE motif like the mucosal HPV E7s (12). MmuPV1 E7, like HPV E7 proteins, also binds to another tumor suppressor, PTPN14, which, in the case of MmuPV1 E7, mediates the virus’ ability to delay epithelial differentiation *in vivo* and which, in the case of HPV E7, promotes basal cell identity *in vitro* (15, 16).

So how does MmuPV1 cause the same types of mucosal cancers in mice as HPVs cause in humans? Could it be that different papillomaviruses use different means to reach the same end? Are there underlying cellular processes that are shared targets of all papillomaviruses that cause cancer, even if not driven by the same biochemical activities? The ability of MmuPV1 to cause mucosal neoplasia raises unique and exciting opportunities to answer these key emerging questions in papillomavirus biology.
